# The Remarkable Antioxidant and Anti-Inflammatory Potential of the Extracts of the Brown Alga *Cystoseira amentacea* var. *stricta*

**DOI:** 10.3390/md19010002

**Published:** 2020-12-23

**Authors:** Gina De La Fuente, Marco Fontana, Valentina Asnaghi, Mariachiara Chiantore, Serena Mirata, Annalisa Salis, Gianluca Damonte, Sonia Scarfì

**Affiliations:** 1 Department of Earth, Environment and Life Sciences (DISTAV), University of Genova, Via Pastore 3, 16132 Genova, Italy; gina.delafuente@edu.unige.it (G.D.L.F.); mapo_95@hotmail.it (M.F.); valentina.asnaghi@unige.it (V.A.); mariachiara.chiantore@unige.it (M.C.); serena.mirata@edu.unige.it (S.M.); 2 Centre of Excellence for Biomedical Research (CEBR), University of Genova, Viale Benedetto XV 9, 16132 Genova, Italy; annalisa.salis@unige.it (A.S.); gianluca.damonte@unige.it (G.D.); 3 Centro 3R, Interuniversitary Center for the Promotion of the Principles of the 3Rs in Teaching and Research, Via Caruso 16, 56122 Pisa, Italy

**Keywords:** brown algae, Fucales, biological effects, gene expression

## Abstract

Inflammation and oxidative stress are part of the complex biological responses of body tissues to harmful stimuli. In recent years, due to the increased understanding that oxidative stress is implicated in several diseases, pharmaceutical industries have invested in the research and development of new antioxidant compounds, especially from marine environment sources. Marine seaweeds have shown the presence of many bioactive secondary metabolites, with great potentialities from both the nutraceutical and the biomedical point of view. In this study, 50%-ethanolic and DMSO extracts from the species *C. amentacea* var. *stricta* were obtained for the first time from seaweeds collected in the Ligurian Sea (north-western Mediterranean). The bioactive properties of these extracts were then investigated, in terms of quantification of specific antioxidant activities by relevant ROS scavenging spectrophotometric tests, and of anti-inflammatory properties in LPS-stimulated macrophages by evaluation of inhibition of inflammatory cytokines and mediators. The data obtained in this study demonstrate a strong anti-inflammatory effect of both *C. amentacea* extracts (DMSO and ethanolic). The extracts showed a very low grade of toxicity on RAW 264.7 macrophages and L929 fibroblasts and a plethora of antioxidant and anti-inflammatory effects that were for the first time thoroughly investigated. The two extracts were able to scavenge OH and NO radicals (OH EC50 between 392 and 454 μg/mL; NO EC50 between 546 and 1293 μg/mL), to partially rescue H_2_O_2_-induced RAW 264.7 macrophages cell death, to abate intracellular ROS production in H_2_O_2_-stimulated macrophages and fibroblasts and to strongly inhibit LPS-induced inflammatory mediators, such as NO production and IL-1α, IL-6, cyclooxygenase-2 and inducible NO synthase gene expression in RAW 264.7 macrophages. These results pave the way, for the future use of *C. amentacea* metabolites, as an example, as antioxidant food additives in antiaging formulations as well as in cosmetic lenitive lotions for inflamed and/or damaged skin.

## 1. Introduction

Inflammation is part of the complex biological response of body tissues to harmful stimuli, such as pathogens, particulate matter, damaged cells, injury or toxic compounds. It develops as a protective response, involving cells of the immune system and a plethora of molecular mediators released by both immune cells and damaged tissues. The function of inflammation is in resolving the initial cause of cell injury, to clear out necrotic cells in the compromised tissues, and to set the right conditions for tissue repair. In some disorders a prolonged inflammation may develop, giving rise to chronic inflammatory diseases, due to the disruption of the molecular signals necessary to control the process which normally is self-limiting. [1]. Furthermore, chronic inflammation is nowadays a broadly recognized feature of aging and of age-related diseases such as diabetes, hypertension, atherosclerosis, and cancer [2]. In particular, the aging phenomenon shows a chronic low-grade inflammation at the systemic level although in the absence of a recognised infection. This phenomenon has been defined as “inflammaging” and represents a significant risk factor for morbidity and mortality in the elderly.

Macrophages play an important role in the inflammatory response, both in acute and chronic inflammation. They act at numerous levels during the response by engulfing foreign agents, by eliminating apoptotic cells and tissue debris in the damaged area. Concomitantly, they release inflammatory mediators able to orchestrate the various phases of the process and the subsequent healing of the tissue. The vast arsenal of released molecules include chemokines, cytokines, growth factors and small second messengers such as nitric oxide (NO) and prostaglandin E_2_ (PGE_2_) produced by overexpressed inducible NO synthase (iNOS), and cyclooxygenase 2 (COX-2) enzymes, respectively [3,4].

Among the cytokines released by activated macrophages, the tumour necrosis factor (TNF)-α, interleukin-1 (IL-1), and interleukin-6 (IL-6) play a prominent role in the propagation and development of the inflammation, together with NO and PGE_2_, by recruiting inflammatory cells and by increasing vascular permeability [5]. The inflammatory response is beneficial if the abovementioned cytokines are produced in appropriate amounts, but it becomes detrimental when they are produced in a deregulated fashion. For instance, the overproduction of IL-1β and TNF promotes an acute systemic inflammatory response typical of multi-organ failure and of septic shock [6]. Therefore, the inhibition of these inflammatory mediators is considered an indispensable approach for the treatment of inflammatory diseases in general.

Inflammation can also be triggered and propagated by oxidative stress, which occurs when the body antioxidant defense system fails to contain the production and release of reactive oxygen species (ROS) [7]. ROS, such as hydrogen peroxide, superoxide, hydroxyl and NO radicals, are generated in many cellular redox processes also during the inflammatory response. The consequence of their overproduction is an uncontrolled reaction with other molecules, such as proteins, DNA and lipids [8], inducing oxidative damage to biomolecules. Therefore, ROS overproduction is detrimental to the body physiological homeostasis, and besides inflammation, it has been shown to participate in the pathogenesis of several human degenerative diseases, including cardiovascular and neurodegenerative disorders and cancer [9,10].

In recent years, due to the numerous diseases in which the oxidative stress is implicated, a great deal of attention from the pharmaceutical industries has arisen for the research and development of new antioxidant compounds. These new compounds have been researched also, and with great success, in marine environment sources. This has allowed the development of new branches of the marine biotechnological research in the quest of natural and safe antioxidative agents from aquatic organisms to replace synthetic antioxidants, some of which have been restricted due to their carcinogenicity [11,12]. In particular, the marine seaweeds have shown the presence of many bioactive secondary metabolites, with great potentialities both from the nutraceutical, as well as from the biomedical point of view [13]. In the last twenty years this research allowed the identification of new antioxidant [14,15], anti-inflammatory [16,17] antimicrobial [15], and anti-tumoral drugs [18] found in red, green and brown macroalgae. Several studies have recognized brown algae as the most powerful source of natural antioxidants compared to green and red algae, mainly because of their remarkable content in polyphenols and phlorotannins [18,19,20,21,22,23,24,25,26]. Large brown algae belonging to the orders Laminariales and Fucales, thrive in the intertidal and subtidal rocky reefs worldwide providing food and refuge for many associated species, as well as controlling nutrient cycling and productivity of their habitats. Therefore, they play a relevant ecological role as foundation species, enhancing habitat complexity, biodiversity and ecosystem functions of rocky coasts [27,28]. These species have shown very high antioxidant activity [25]. In the Mediterranean Sea the most important foundation species belong to the widespread genus of *Cystoseira sensu lato* (Fucales order), which holds very promising features in terms of secondary metabolites production [29,30,31,32,33]. In fact, many interesting molecules such as terpenoids, alkaloids and steroids have been isolated from different species of the genus, but few studies on the pharmacological properties of these compounds have been published [34,35,36,37,38,39,40,41,42,43].

In this study, the properties of 50%-ethanolic and DMSO extracts from the species *C. amentacea* var. *stricta* Montagne (hereafter *C. amentacea*) were thoroughly investigated for the first time in seaweeds collected in the Ligurian Sea (north-western Mediterranean). Since in the literature there is poor knowledge on the molecular targets of the compounds contained in *C*. *amentacea* extracts, the bioactive properties of these extracts were assessed in terms of quantification of specific antioxidant activities by opportune ROS scavenging tests, identifying the main oxidant targets of the extracts inside the cells and the anti-inflammatory properties in LPS-stimulated macrophages by evaluating the specific abatement of dangerous inflammatory cytokines and mediators by molecular studies. Contextually, cytotoxicity was also assessed in order to investigate the possible use of these extracts as nutraceuticals, cosmeceuticals or anti-inflammatory drugs in human health issues.

## 2. Results and Discussion

### 2.1. Extraction Yield, Total Phenolic and Flavonoid Content of C. amentacea Extracts

The 50%-ethanol and DMSO extractions used to retrieve a more hydrophilic and a more hydrophobic fraction, respectively, were performed in mild conditions (stirring at 30 °C for 48 h, in the dark) resulting in a yield of 310 ± 50 and 171 ± 32 mg of crude extract per gram of dried algae, respectively. These gentle conditions were chosen with the aim of recovering molecular products with the maximal stability, since it has been reported [40] that some degree of thermal instability (15–30% depending on the molecular species) is observed for polyphenols at temperatures higher than 60°C, as well as a certain degree of photo-oxidation by UV-light exposure (10–50%). These yields were comparable to the yields obtained by various extraction solvents used to assess the bioactive potential of *C. barbata* by Trifan et al. [41], and significantly higher than those obtained by Ruberto et al. [36] by CH_2_Cl_2_ organic extraction of various species from the genus *Cystoseira* (from 2.1% to 6.7% depending on the species). In particular, the ethanolic extract in our study (31% yield) was comparable to the 70%-acetone extract for *C. barbata* by Trifan et al. (24% yield) [41], although in our case it was obtained by using a cheaper and less toxic solvent. A great contributing factor to the antioxidant and anti-inflammatory activity of numerous plant extracts is due to their content in polyphenols. Therefore, we initially measured the total phenolic content (TPC) and the total flavonoid content (TFC) of the two extracts by the appropriate colorimetric assays. The results, expressed as μg of TPC/TFC per mg of crude extract, are displayed in Table 1. Both extracts contained measurable quantities of TPC and TFC, although in different proportions. The ethanolic extract in fact, contained 20.3 μg TPC/mg crude extract (corresponding to 6.3 mg/g dried algae) of which the 15.3% was constituted by flavonoids, while in the DMSO extract the TPC content was 65.9 μg/mg crude extract (corresponding to 11.2 mg/g dried algae) of which the 24% was represented by flavonoids. Both quantifications underlined a higher content of polyphenols as well as of flavonoids in the DMSO extract with respect to the 50%-ethanol extract. In particular, the TPC and TFC contents of the DMSO extract were 3.2 and 5.1 folds higher than the ethanolic extract, respectively. These results are very similar to the TPC observed in the single other study on *C. amentacea* collected from the Mediterranean Sicilian coastal area evaluating the seasonal fluctuation of TPC in this seaweed [37]. In that case, extraction of polyphenols was obtained with 95% ethanol and the TPC displayed a seasonal fluctuation from a minimum TPC content in winter (less than 1 mg/g dried algae) to a maximum content in summer (6 mg/g dried algae). Since the algae used in our study were collected in the summer season in the Mediterranean from the Ligurian coasts, our data confirm the data from the Sicilian study. Furthermore, the results of the two studies indicate that there is no difference in terms of TPC yield between the two extraction methods (50%-ethanol or 95%-ethanol), although the conditions used in our study are more attractive both for their safety (less solvent flammability) and their convenience (cheaper solution). Comparable to the values obtained in *C. amentacea* from the Mediterranean Sea, were also the TPC values obtained from Kosanic et al. [38] from the same species collected in the Adriatic Sea and acetone-extracted, which phenolic content was of 81 μg/mg extract. Other studies report TPC yields from extracts of various species of the *Cystoseira* genus, displaying a large variability: 50–61 mg/g dried weight in the aqueous extracts from *C. compressa*, *C. crinita* and *C. sedoides* (Mediterranean Tunisian coastal areas) in Mhadhebi et al. [34], 126–236 mg/g dried weight in the acetone, methanol and water extracts from *C. barbata* (Black Sea coastal areas) in Trifan et al. [41], 4.7–32 mg/g dried weight from *C. humilis*; 45–165 mg/g dried weight from *C. tamariscifolia* and 17–127 mg/g dried weight from *C. usneoides* (Portuguese coasts) in organic sequential extracts, in Vizetto-Duarte et al. [39]. Comparing *C. amentacea* TPC content to the other *Cystoseira* species analysed in the literature we can infer that this species seems in general to a have a lower content in TPC compared to the others (1 order of magnitude), with similar yields obtained only in *C. humilis* from the Portuguese coasts. This is probably not only due to the different approach applied in the various studies, i.e., extraction conditions and seasonality, but also to other factors related to the variability of the habitats of each species, such as irradiance, temperature and bathymetric level.

### 2.2. HPLC-MS Analysis

**Figure 1:**
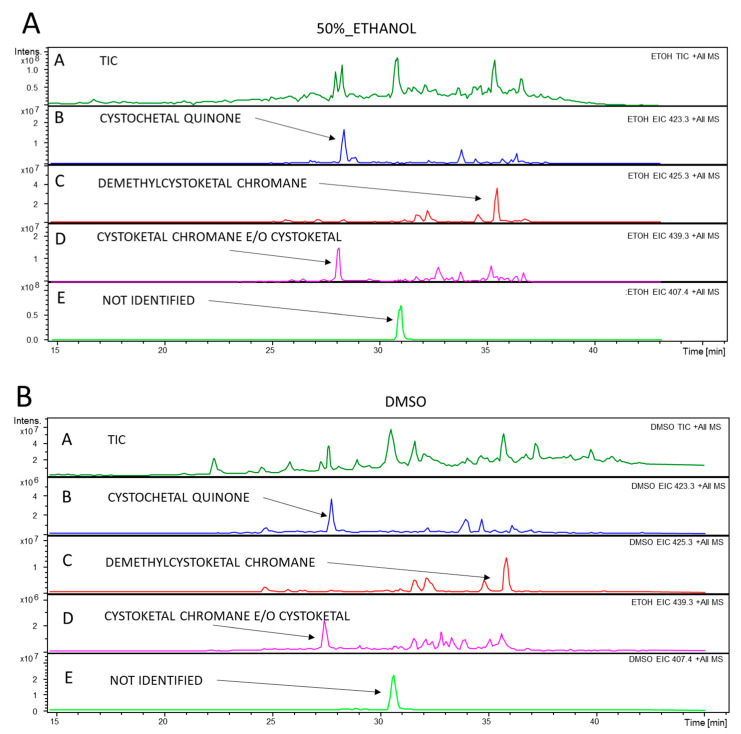
Total ion current (TIC) and Extracted ion chromatograms obtained by full scan MS/MS analysis coupled to HPLC separation of an aliquot of the *C*. *amentacea* 50% ethanol extract in panel (**A**) and DMSO extract in panel (**B**) (starting dilution 5 mg/mL), acquiring the most abundant species under each peak. Chromatogram A in both panels represent the TIC of each extract while chromatograms B, C, D and E in both panels represent the extracted ion chromatograms of 423.3 (B), 425.3 (C) 439.3 (D), and 407.4 (E) mass/charge ratios. Acquisition was performed on negative and positive ions in the 100–1000 mass range and analyzed using the integrated software.

**Figure 2:**
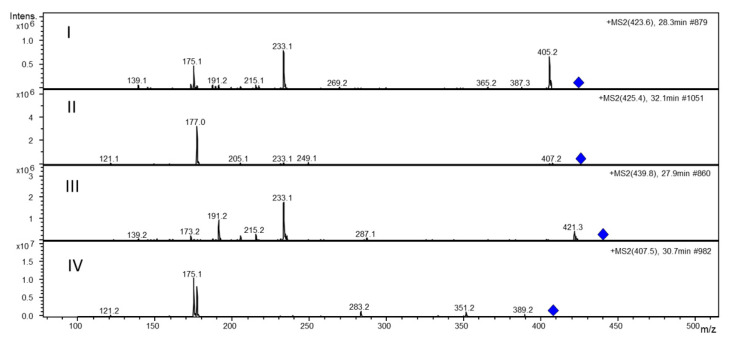
Tandem mass fragmentation spectra of extracted ions (blue diamonds) reported in Figure 1 (see chromatograms B, C, D and E, respectively, of both panels) of 423.3 (panel **I**), 425.3 (panel **II**), 439.3 (panel **III**) and 407.4 (panel **IV**) mass/charge ratios obtained by full scan MS/MS analysis coupled to HPLC and performed as reported in Figure 1.

The HPLC-MS/MS analysis was performed with the aim of identifying the main molecular components present in the extracts. The analysis of the two extracts showed the same molecular species in both samples and mainly pointed out the presence of meroditerpene-like structures previously described in the same genus [26,29,30,31,32]. In Figure 1 the tandem MS analyses of the 50%-ethanol extract (panel A) and of the DMSO extract (panel B) are shown. The figure shows for both extracts the Total Ion Chromatogram (TIC, chromatogram A in both panels), and then the fragmentation patterns of four different meroditerpene molecules found in both extracts (in both panels, chromatograms B, C, D and E). These are ascribable to structures containing the chroman or quinone groups as: cystoketal quinone (chromatogram B in both extract), demethylcystoketal chromane (chromatogram C in both extracts) and cystoketal chromane, and/or cystoketal (chromatogram D in both extracts) highlighted in the figure as extracted ion chromatograms of respectively, 423.3 (B) and 425.3 (C) and 439.3 (D) mass/charge ratios. Figure 2 shows the tandem mass fragmentation spectra (panels I, II, III and IV) of the ions extracted in Figure 1 (chromatograms B, C, D and E, respectively in both extracts) confirming the structure of the molecules.

Another unidentified signal at *m*/*z* 407.4 (Figure 1, chromatogram E in both extracts) is also present. In Figure 2, panel IV, the relative tandem mass spectrum is reported, where the simultaneous presence of the 175 and 177 fragments is attributable to both the chroman and the quinone reduced form (hydroquinone). In fact, the *m*/*z* 175 is consistent only with the quinone nucleus, while the *m*/*z* at 177 is imputable to the presence of both the hydroquinone and the chroman group. Besides, the presence of the *m*/*z* 191 fragment is related to methyl derivatives of either these nuclei. In conclusion, the HPLC-MS analysis points out that both extracts seem to retrieve the same meroditerpene class of molecules from the algae, confirming the abundance of these bioactive compounds [26,32] in the *Cystoseira amentacea* Mediterranean seaweed. Therefore, the different efficacy of the two extracts in the various antioxidant and anti-inflammatory tests may be attributable to a quantitative, and not qualitative, difference of the molecules contained. This is also deductible from the TPC quantification reported in Table II, where, as already commented, the DMSO extract show a phenolic content almost three times higher than the 50%-ethanol extract, which could explain the general better performance of the first one upon the second in the various tests performed in this study.

### 2.3. Antioxidant Activity Evaluation of C. amentacea Extracts

**Figure 3:**
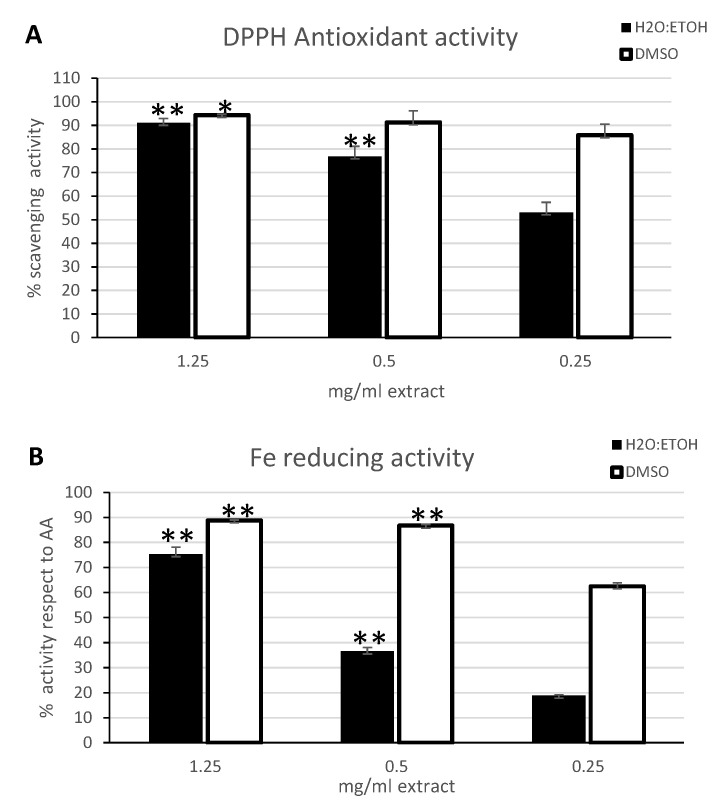
Activity and Fe reducing power of *C. amentacea* extracts in spectrophotometric tests. (**A**) ROS scavenging activity by the DPPH assay. Data are the mean ± S.D. of three experiments performed in duplicate and are expressed as percentage of antioxidant activity respect to the absorbance of the negative control (calculated as specified in Section 4.7). Black bars: 50%-ethanol extract, white bars: DMSO extract. Asterisks indicate significance in paired Tukey test between the various concentrations used (ANOVA *p* < 0.0005, black bars: Tukey between 1.25 and 0.5, between 1.25 and 0.25, and between 0.5 and 0.25 ** *p* < 0.001; white bars: Tukey between 1.25 and 0.5, and between 1.25 and 0.25, * *p* < 0.005) (**B**) Fe (III) reducing power assay measured by the potassium ferricyanide method. Data are the mean ± S.D. of three experiments performed in duplicate and are expressed as percentage of reducing power respect to the ascorbic acid positive control (calculated as specified in Section 4.8). Black bars: 50%-ethanol extract, white bars: DMSO extract. Asterisks indicate significance in paired Tukey test between the various concentrations used (ANOVA *p* < 0.00001, black bars: Tukey between 1.25 and 0.5, between 1.25 and 0.25, and between 0.5 and 0.25 ** *p* < 0.0005; white bars: Tukey between 1.25 and 0.5, between 1.25 and 0.25, and between 0.5 and 0.25 ** *p* < 0.0005).

The antioxidant activity of the two extracts was evaluated by different methods investigating the overall radical scavenging activity and the Fe reducing power of *C. amentacea* metabolites, as well as the scavenging capacity of biologically dangerous radicals such as the highly reactive nitric oxide (NO) and hydroxyl radicals (OH). The range of concentrations used for the assays was chosen to allow a comparison with previous works [34,35,36,39,41,42,43]. The overall radical scavenging activity measured by the DPPH assay revealed an elevated potential for both extracts (Figure 3A). At the highest concentration tested, in fact, both extracts showed a scavenging potential higher than 90%, while at the lowest concentration the ethanolic extract retained a scavenging activity slightly higher than 50% (black bars), with a calculated EC_50_ of 205.1 μg/mL, while at the same concentration the DMSO extract (white bars) showed a scavenging activity still higher than 80%, with a calculated EC_50_ of 0.34 μg/mL. Similar to our data, also the acetone extracts of *C. amentacea* var. *spicata* (Adriatic Sea, Montenegro coasts) from Stanojkovic et al. [35] showed a DPPH EC_50_ of 150 μg/mL with an antioxidant activity close to the ethanolic extracts obtained in our study, indicating that the abundance of antioxidants metabolites produced by the same species (although a different variety) does not change considerably between the two marine environments (North-western Mediterranean and Adriatic). Conversely, although the TPC content is usually much lower than other species of the *Cystoseira* genus, *C. amentacea* var. *stricta* is considerably richer in antioxidant compounds or containing comparable amounts, depending on the species and on the extraction procedure. For example, Trifan et al. [41] reported a DPPH EC_50_ of *C. barbata* extracts closely comparable to our study (from 88 to 211 μg/mL depending on the extraction solvent); Mhadhebi et al. [34] documented DPPH EC_50_ values of 12, 20, and 75 μg/mL for *C. compressa*, *C. crinita* and *C. sedoides* extracts, respectively, while Vizetto-Duarte et al. [39] obtained significantly lower antioxidant potential from the organic extracts of *C. humilis* and *C. usneoides* (DPPH EC_50_ > 1 mg/mL) compared to *C. tamariscifolia* extract (DPPH EC_50_ between 170 and 1080 μg/mL). Particularly, the latter is the only one with values comparable to *C. amentacea* var. *stricta* extracts obtained in our study. Values comparable with our results were also obtained by Andrade et al. [42] for *C. tamariscifolia* and *C. usneoides* ethanolic extracts, while a very low antioxidant potential was found in *C. nodicaulis* and in *C. spongiosus* (DPPH EC_50_ > 1 mg/mL). Finally, Belattmania et al. [43] fractionation of fatty acids from *C. humilis* revealed a DPPH EC_50_ of 580 μg/mL, indicating that the antioxidant potential of the *Cystoseira* genus metabolites resides also in other promising classes of compounds besides the typical phenols. 

### 2.4. Scavenging Properties of C. amentacea Extracts

After assessing the overall antioxidant activity of the extracts, the specific scavenging properties of particularly dangerous reactive oxygen species were analyzed in order to understand which of them would be intracellularly mostly affected by the pharmacological use of *C. amentacea* extracts, since usually, ROS are produced under oxidative stress as well as during acute and chronic inflammation.

The Fe-reducing power assay, was performed by the potassium ferricyanide method and is another measure of the antioxidant power of the extract, furthermore the ability to reduce Fe to the ferrous form can be also important in preventing the Fenton reaction, leading to the production of superoxide anion and may also facilitate the removal of hydroxyl radical by Fe(II) oxidation [44]. This assay revealed the presence of this chemical property in the two *C. amentacea* extracts (Figure 3B), showing significantly higher capacity to reduce the Fe(III) ion to Fe(II) in the DMSO extract (white bars) compared to the ethanolic extract (black bars). In particular, the results are expressed as percentage of the reducing activity of the two extracts at different concentrations compared to the reducing activity of a concentration of ascorbic acid that allows the complete Fe reduction (20 μg/mL, in our experimental conditions). The DMSO extract showed a reducing activity close to 90% at the highest concentration and higher than 60% at the lowest concentration (EC_50_ 113.9 μg/mL). Conversely, the highest concentration of the ethanolic extract exerted a reducing activity of 75%, while at the lowest concentration the activity was less than 20% (EC_50_ 643.5 μg/mL). The study of Mhadhebi et al. [34] is the only one that reports this specific assay on *Cystoseira* species extracts, particularly on *C. compressa*, *C. crinita* and *C. sedoides* whose values were 2.6, 0.9 and 0.7, respectively, expressed as mg of gallic acid equivalents (GAE)/g of dried alga. If we express our results on *C. amentacea* extracts as mg of ascorbic acid equivalents (AAE)/g of dried algae we obtain values of 2.5 and 3.7 mg AAE/g dried algae, for ethanolic and DMSO extracts, respectively. Since it has been reported that gallic acid and ascorbic acid share very similar antioxidant activities [45] we can deduce that the performance of *C. amentacea* extracts in the Fe-reducing activity is comparable or higher respect to the activities reported in the literature for *C. compressa*, *C. crinita* and *C. sedoides*, holding very promising results.

**Figure 4:**
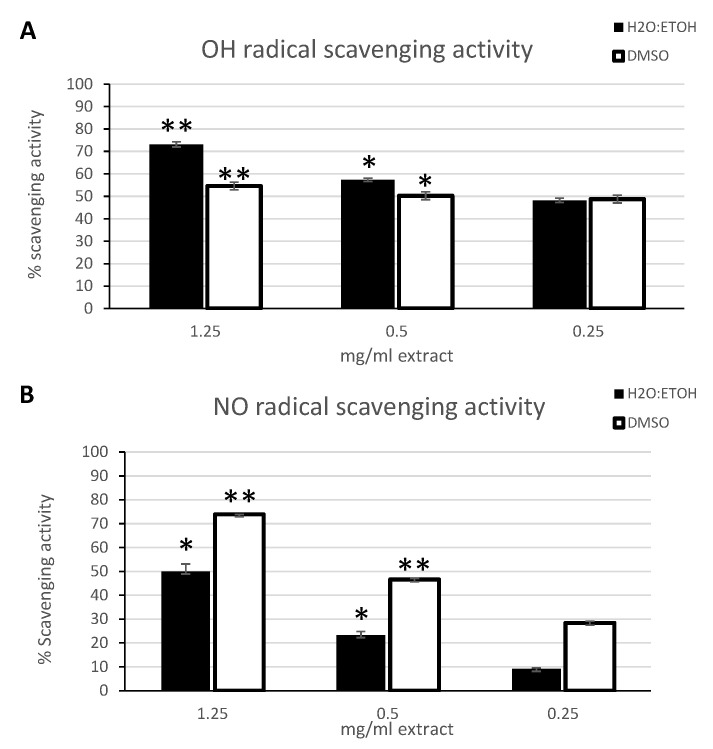
OH and NO radical scavenging activity of *C. amentacea* extracts in spectrophotometric tests. (**A**) OH radical scavenging activity by the Mohr’s salt assay. Data are the mean ± S.D. of three experiments performed in duplicate and are expressed as percentage of antioxidant activity respect to the absorbance of the negative control (calculated as specified in Section 4.9). Black bars: 50%-ethanol extract, white bars: DMSO extract. Asterisks indicate significance in paired Tukey test between the various concentrations used (ANOVA *p* < 0.001, black bars: Tukey between 1.25 and 0.5, between 1.25 and 0.25 ** *p* < 0.01, between 0.5 and 0.25 * *p* < 0.05; white bars: Tukey between 1.25 and 0.5, between 1.25 and 0.25, ** *p* < 0.01, between 0.5 and 0.25 * *p* < 0.05) (**B**) NO radical scavenging activity measured by the sodium nitroprusside method coupled to the Griess assay. Data are the mean ± S.D. of three experiments performed in duplicate and are expressed as percentage of antioxidant activity respect to the absorbance of the negative control (calculated as specified in Section 4.10). Black bars: 50%-ethanol extract, white bars: DMSO extract. Asterisks indicate significance in paired Tukey test between the various concentrations used (ANOVA *p* < 0.0001, black bars: Tukey between 1.25 and 0.5, between 1.25 and 0.25, and between 0.5 and 0.25 * *p* < 0.001, white bars: Tukey between 1.25 and 0.5, between 1.25 and 0.25, and between 0.5 and 0.25 ** *p* < 0.0001).

OH and NO radicals are responsible for a broad molecular damage inside and outside the cells during oxidative stress and they are also involved as second messengers in key transduction pathways propagating the inflammatory signals [46]. Therefore, the specific scavenging capacity of the *C. amentacea* extracts towards the two radical species was tested by the respective spectrophotometric assays. The results demonstrate, in this case, that an elevated and selective scavenging potential of the ethanolic and DMSO extracts is detected (Figure 4). In particular, the OH radical scavenging activity was tested by the Mohr’s salt assay showing a higher scavenging activity for the ethanolic extract compared to the DMSO extract (Figure 4A, black bars versus white, respectively) at the highest concentrations, with similar values at the lowest concentration tested. In fact, at the highest concentration the ethanolic extract exhibited a scavenging activity slightly above 70% while at the lowest concentration it was 42% (EC_50_ 292.9 μg/mL). Conversely, the DMSO extract showed a scavenging activity of 54.5% at the highest concentration tested and of 48.7% at the lowest concentration (EC_50_ 454.5 μg/mL). The study of Andrade et al. [42] is, to our knowledge, the only one that reports of this assay on the *Cystoseira* genus, documenting EC25 values of hydroxyl radical scavenging below 500 μg/mL, i.e., comparable to the *C. amentacea* values of our study, for the *C. tamariscifolia* species (210 μg/mL), while for the other species analyzed (*C. usneoides*, *C. nodicaulis* and *C. spongiosus*), a significantly poorer scavenging activity was shown. Thus, to date, we can assess that, according to literature, the *C. amentacea* extracts show the highest OH radical scavenging activity among the *Cystoseira* genus.

Finally, the NO scavenging activity of the extracts was evaluated by the nitroprusside NO donor assay. In this case, the DMSO extract showed a higher scavenging potential compared to the ethanolic extract (Figure 4B, white bars vs. black, respectively). In particular, the DMSO extract, at the highest concentration, showed a NO scavenging potential higher than 70%, while at the lowest concentration it was slightly below 30% (EC_50_ 546.2 μg/mL). Instead, the ethanolic extract exhibited a scavenging activity of 49.9% and 9.1% at the highest and lowest concentration, respectively (EC50 1293 μg/mL). Compared to the only study of Andrade et al. [42] in literature, as abovementioned, the values obtained with *C. amentacea* in our study resulted, also in this case, similar to *C. tamariscifolia* (EC_25_ of 240 μg/mL) and *C. nodicaulis* (EC_25_ 480 μg/mL), but higher than *C. usneoides* (EC_25_ 790 μg/mL) and *C. spongiosus* (EC_25_ 3190 μg/mL).

Overall, these data indicate that the antioxidant potential of *C. amentacea* var. *stricta* can be considered one of the most promising among the species of the *Cystoseira* genus with documented activity. This activity cannot be solely ascribed to the TPC and TFC content of the ethanolic and DMSO extracts because, as reported in the previous section, the TPC content in *C. amentacea* var. *stricta* extracts is, on the average, one order of magnitude lower than those measured in the other species of the *Cystoseira* genus. An important contributing factor to this notable antioxidant activity is likely due to the very rich variety and abundance of chemical products that have been identified in the various species of the genus, from terpenoids (meroditerpenes, linear diterpenes), also identified in our HPLC/MS analysis (Figure 1 and Figure 2), to carbohydrates, lipids and vitamins to which the radical scavenging activity can also be ascribed [32]. Therefore, since for *Cystoseira amentacea* var. *stricta* few dated studies report of its phytochemical features [29,30,31,47,48], further studies could be performed, in order to have a clearer picture of the variety of compounds produced by this species.

### 2.5. Cytotoxicity Test of C. amentacea Extracts

**Figure 5:**
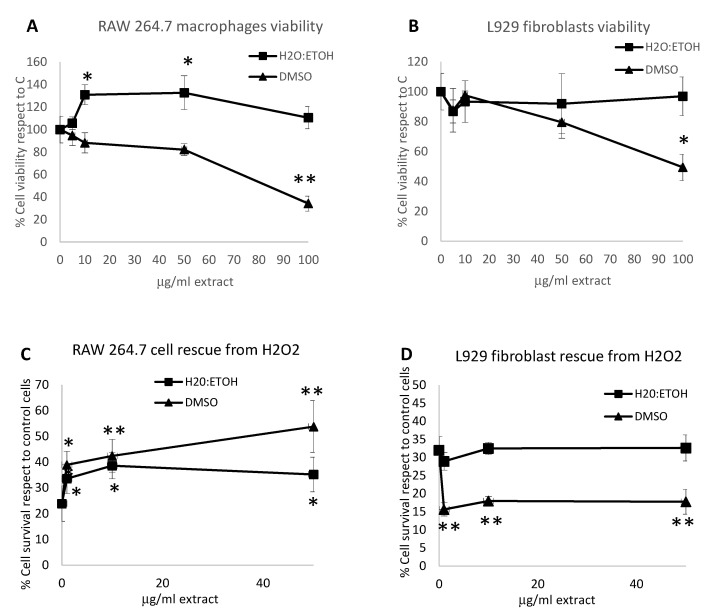
Toxicity evaluation. (**A**) RAW 264.7 cell growth quantitative evaluation, by the cell viability MTT test at 24 h, in the presence of increasing concentrations of *C. amentacea* 50%-ethanol (black square indicator) and DMSO (black triangle indicator) extracts. Results are expressed as cell percentage respect to control, untreated cells (in presence of vehicle) and are the mean ± S.D. of three experiments performed in quadruplicate. Asterisks indicate significance in paired Tukey test (ANOVA, *p* < 0.05; Tukey vs. C: * *p* < 0.05, ** *p* < 0.001, respectively). (**B**) L929 fibroblast cell growth quantitative evaluation, in the same conditions as (**A**). Black square indicator: 50%-ethanol extract, black triangle indicator: DMSO extract. The results are expressed as cell percentage respect to control, untreated cells and are the mean ± S.D. of three experiments performed in quadruplicate. Asterisks indicate significance in paired Tukey test (ANOVA, *p* < 0.05; Tukey vs. C: * *p* < 0.005, respectively). (**C**) Cell death rescue from 500 μM H_2_O_2_-challenge of RAW 264.7 macrophages in presence of 50%-ethanol (black square indicator) and of DMSO (black triangle indicator) extracts, evaluated by MTT test at 24 h. The results are expressed as cell percentage respect to control, untreated cells and are the mean ± S.D. of two experiments performed in quadruplicate. Asterisks indicate significance in paired Tukey test (ANOVA, *p* < 0.05; Tukey vs. H_2_O_2_: * *p* < 0.05, ** *p* < 0.01, respectively). (**D**) Cell death rescue from 500 μM H_2_O_2_-challenge of L929 fibroblasts, in the same conditions as (**A**). Black square indicator: 50%-ethanol extract, black triangle indicator: DMSO extract. Results are expressed as cell percentage respect to control, untreated cells and are the mean ± S.D. of two experiments performed in quadruplicate. Asterisks indicate significance in paired Tukey test (ANOVA, *p* < 0.05; Tukey vs. H_2_O_2_: ** *p* < 0.005).

The cytotoxicity of the two extracts at various concentrations was evaluated in two cell lines to investigate the possible use of these extracts as nutraceuticals or anti-inflammatory drugs in human health issues. The macrophage cell line RAW 264.7 and the fibroblast cell line L929 were incubated for 24 h with various dilutions of the two extracts (from 5 to 100 μg/mL) and then the cell viability was evaluated by the MTT test and compared to untreated, control cells (Figure 5A and B, respectively). The results showed that, for both cell lines, the ethanolic extract never affected the cell viability at all concentrations tested (Figure 5A, RAW 264.7 macrophages; Figure 5B L929 fibroblasts; square indicator, respectively). The ethanolic extract also showed a slight cell number increase at 10 and 50 μg/mL concentration (*p* < 0.05 for both compared to control) indicating that this extract may be safely used in humans. Conversely, the DMSO extract showed a significant impairment of cell viability at 24 h, but only at the highest concentration (100 μg/mL), in both cell lines (66% mortality in RAW 264.7 macrophages, *p* < 0.001 compared to C, and 50% cell mortality in L929 fibroblasts; triangle indicator, *p* < 0.005 compared to the control, respectively). At concentrations ≤ 50 μg/mL the DMSO extract could be also considered reasonably safe in both cell lines (Figure 5A and B, triangle indicator, *p* < 0.084 and *p* < 0.15 for 50 μg/mL compared to control in each cell line, respectively). Our results differ from those reported by Stanojkovic et al. [35] on *C. amentacea* extracts from the Adriatic Sea, showing a significant cytotoxic potential on different tumor cell lines (EC_50_ < 30 μg/mL in human breast cancer cells, and EC_50_ < 100 μg/mL in human cervix and human colon carcinomas). This discrepancy can be ascribed to the different conditions used by Stanojkovic et al. where a Soxhlet extractor with acetone as solvent was used, retrieving a more hydrophobic fraction of algal metabolites, with respect to our mild extraction conditions, and this could explain the different results of the Ligurian Sea species compared to those from the Adriatic Sea. Indeed, a certain degree of toxicity was also observed in our DMSO extracts, retrieving a more organic fraction and, consequently closer to the acetone extraction of the group of Stanojkovic. In our conditions in fact, we obtained EC50 values of 83 and 103.6 μg/mL for RAW 264.7 macrophages and L929 fibroblasts, respectively. A significant cytotoxic activity in organic extracts has also been demonstrated in other *Cystoseira* species, as for instance, in *C. tamariscifolia* hexane and diethylether extracts [39] that showed an EC_50_ <30 μg/mL on tumor cell lines, and also in *C. barbata* acetone extracts [41] that showed an EC_50_ <100 μg/mL on MCF7 mammary adenocarcinoma cells. Overall, our results demonstrate that the ethanolic and DMSO extracts from *C. amentacea*, that was collected in the Ligurian Sea, could be safely used as nutraceuticals or cosmeceuticals since they have revealed no grade, or extremely low grade of toxicity and a potent antioxidant activity. These features may be exploited for the formulation of new biologically active additives in dietary supplements, for instance in the elderly, or, in cosmetic products for skin treatments, where the antioxidants exert significant anti-aging effects. Indeed, besides the antioxidant and anti-inflammatory potential demonstrated in our study, the *Cystoseira sensu lato* extracts display other important features exploitable as ingredients in functional foods that have been already disclosed in previous studies, such as (i) in vivo antidiabetic properties of lipid and phenolic extracts [49], (ii) in vivo liver protection, inhibition of lipase activity and body weight lowering of sulphate polysaccharide extracts [50] and (iii) high PUFA/FA ratio in *Cystoseira* fatty acid content, with consequent low atherogenic and thrombogenic indexes [43,51]. Overall, these features indicate a high nutraceutical value of the algal extracts that could be realistically exploited by the industry. Finally, in addition to the antioxidant and anti-inflammatory properties, the cosmetic use of *Cystoseira* extracts for lenitive skin concoctions would take advantage of a documented antimicrobial activity, indicating that the extracts could exert a certain grade of protection from skin infections [38,43] and also an anti-hyaluronidase activity [52] that could help in the maintenance of dermal thickness, which thinning is one of the most important negative aspects of skin aging.

### 2.6. Cell Death Rescue and Intracellular ROS Scavenging of C. amentacea Extracts

Since in our conditions, the ethanolic and DMSO extracts were not toxic at 100 μg/mL and 50 μg/mL, respectively, these values were the respective highest concentrations, used in the following experiments. In detail, the effectiveness of the antioxidant and anti-inflammatory activity of the two extracts in in vitro cellular models of toxicity and inflammation was evaluated. The rescue from H_2_O_2_-induced cell death was evaluated in both RAW 264.7 macrophages and L929 fibroblasts in the presence of the two extracts after 24 h strong oxidant challenge with 500 μM H_2_O_2_ (Figure 5C and D, respectively). In the RAW 264.7 cell line after H_2_O_2_ treatment only a 23.9% cell survival was observed compared to control, which was only slightly higher in the ethanolic extracts treated cells (Figure 5C; 35.2 at 50 and 38.6% at 10 μg/mL, square indicator, *p* < 0.05 for both extracts, compared to H_2_O_2_ treatment) and significantly higher in the DMSO extracts (Figure 5C; 53.8 at 50 and 42.5% at 10 μg/mL, triangle indicator, *p* < 0.01 for both extracts compared to H_2_O_2_ treatment). On the contrary, in the L929 fibroblast cell line, where H_2_O_2_ treatment, compared to the control lead to a 32% cell survival, it was never possible to observe a beneficial effect in cell viability in the presence of the two extracts at all concentrations tested (Figure 5D), indicating a certain variability in the effects of the *C. amentacea* extracts towards different cell types. Notably, macrophages with respect to fibroblasts are highly reactive immune cells, which undergo a rapid functional production of ROS upon activation by pro-inflammatory stimuli. Therefore, the positive results on cell rescue of H_2_O_2_-challenged RAW 264.7 macrophages seem more meaningful for the possible use of the extracts as anti-inflammatory drugs, since ROS production by macrophages is indeed physiologically more relevant, more frequent and more dangerous than in other cell types in the human organism.

**Figure 6:**
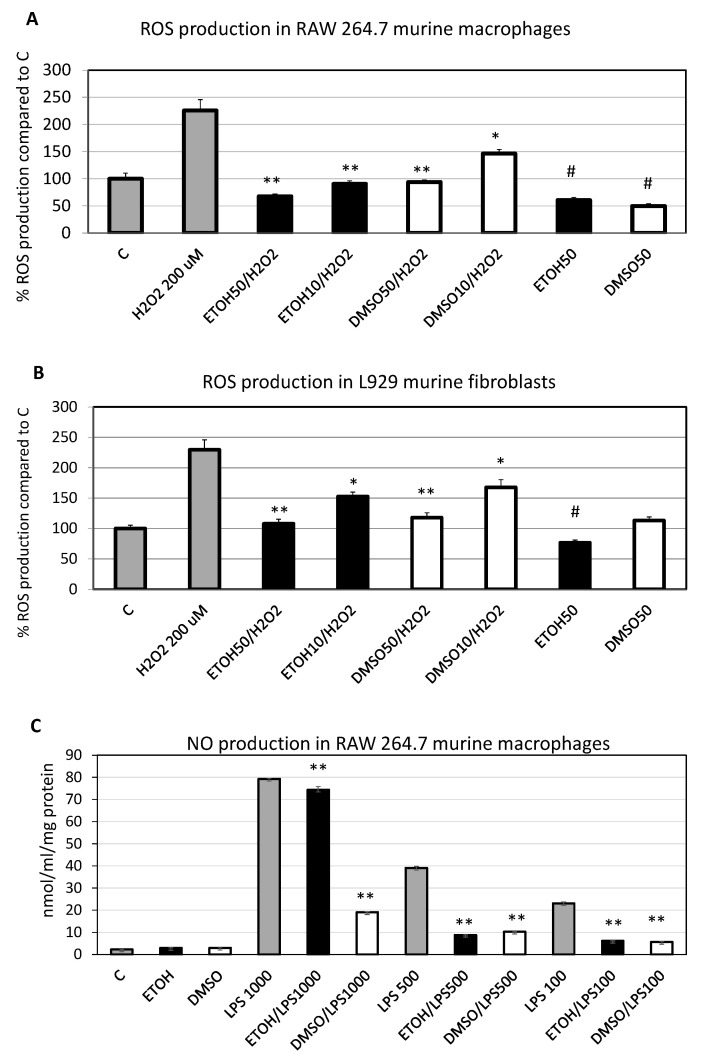
*amentacea* extract ROS and NO scavenging activity in cellular assays. (**A**) Intracellular ROS production measured by DCF fluorometric analysis in RAW 264.7 murine macrophages incubated for 2 h with 200 μM H_2_O_2_ (positive control) in presence or absence of 50 and 10 μg/mL of 50%-ethanol (black bars), or DMSO (white bars) extracts, respectively. The results are expressed as percentage of ROS production respect to control, untreated cells (in presence of vehicle) and are the mean ±SD of three experiments performed in quadruplicate. Asterisks indicate significance in Tukey test (ANOVA *p* < 0.0001; Tukey vs. H_2_O_2_, * *p* < 0.05, ** *p* < 0.005, respectively; Tukey vs. C # *p* < 0.05). (**B**) Intracellular ROS production in L929 cells in the same conditions as (**A**). Results are expressed as percentage of ROS production respect to control, untreated cells and are the mean ±SD of three experiments performed in quadruplicate. Asterisks indicate significance in Tukey test (ANOVA *p* < 0.00001; Tukey vs. H_2_O_2_, * *p* < 0.005, ** *p* < 0.0001, respectively; Tukey vs. C # *p* < 0.05). (**C**) Quantification by Griess assay of NO production in the cell medium of RAW 264.7 macrophages stimulated for 24 h by increasing concentrations of LPS (grey bars, 100-500-1000 ng/mL) in the presence of 100 μg/mL 50%-ethanol (black bars) or 50 μg/mL DMSO (white bars) extract. Results are expressed as nmol of NO production/mL medium/mg total cellular protein and are the mean ±SD of three experiments performed in duplicate. Asterisks indicate significance in Tukey test (ANOVA *p* < 0.00001; Tukey vs. the respective LPS concentration ** *p* < 0.0001).

To evaluate the anti-inflammatory potential of *C. amentacea* extracts, the inhibition of the respiratory burst measured by quantification of intracellular ROS production after H_2_O_2_ challenge in both RAW 264.7 macrophages and L929 fibroblasts (Figure 6A and B, respectively) was quantified.

The quantification of intracellular ROS production after 2 h of 200 μM H_2_O_2_ stimulation revealed that the percentage of ROS production in RAW 264.7 cells was 225%, compared to the control cells (Figure 6A, “H2O2 200 μM” bar vs. “C” bar, *p* < 0.01). This production was completely inhibited by the ethanolic extract at both concentrations tested, namely 50 and 10 μg/mL (Figure 6A, “ETOH50/H2O2” and “ETOH10/H2O2” bars, *p* < 0.005 for both, compared to “H2O2 200 μM”, respectively). Instead, the inhibition in presence of the DMSO extract was complete only at the highest concentration tested (50 μg/mL, “DMSO50/H2O2” *p* < 0.005 compared to “H2O2 200 μM”) and partial at the lowest (10 μg/mL, “DMSO10/H2O2” bar, 63% inhibition respect to “H2O2 200 μM” bar, *p* < 0.05). Furthermore, administration of the two extracts alone was able *per se* to significantly lower also the basal ROS production in RAW 264.7 macrophages (40% inhibition for “ETOH50” and 51% inhibition for “DMSO50” bars, compared to control, *p* < 0.05 for both). The same experiment was performed in L929 fibroblasts and, also in this case, inhibition of intracellular ROS production after H_2_O_2_ administration to cells in the presence of the two extracts was obtained, showing a lower efficiency compared to RAW 264.7 macrophages (Figure 6B vs. A, respectively). Similarly to macrophages, H_2_O_2_-stimulated fibroblasts after 2 h raised the intracellular ROS production to 230% compared to control cells (Figure 6B, “H2O2 200 μM” bar vs. “C” bar, *p* < 0.0001) and this overproduction was completely suppressed by both extracts at the highest concentration of 50 μg/mL (Figure 4B, “ETO50/H2O2” and “DMSO50/H2O2” bars, respectively, *p* < 0.0001 for both compared to “H2O2 200 μM”), while only partially at the lowest concentration (60% for “ETOH10/H2O2” bar and 48% for “DMSO10/H2O2” bar respect to “H2O2 200 μM” bar, *p* < 0.005 for both). Finally, only the ethanolic extract per se was able to slightly diminish intracellular basal ROS production in L929 fibroblasts respect to control cells by 24% (“ETOH50” bar vs. “C”, *p* < 0.05). Stanojkovic et al. [35] report that *C. amentacea* acetone Soxhlet extracts also showed a partial decrease of ROS production by H_2_O_2_-challenged erythrocytes and polymorphonuclear granulocytes but, in this case using different types of cells and performing a qualitative assessment, no precise quantification of the intracellular ROS inhibition was performed. Therefore, to our knowledge, the present study reports, for the first time, a remarkable intracellular antioxidant potential of the extracts from an alga belonging to the genus *Cystoseira*, indicating that the secondary metabolites purified by our ethanolic and DMSO extracts are able to cross cell membranes and act at the level of the cytoplasm. This is a major finding because it clearly indicates the effectiveness of these extracts as potential drugs in human cells when the physiological oxidative balance is impaired.

### 2.7. Anti-Inflammatory Potential of C. amentacea Extracts

**Figure 7:**
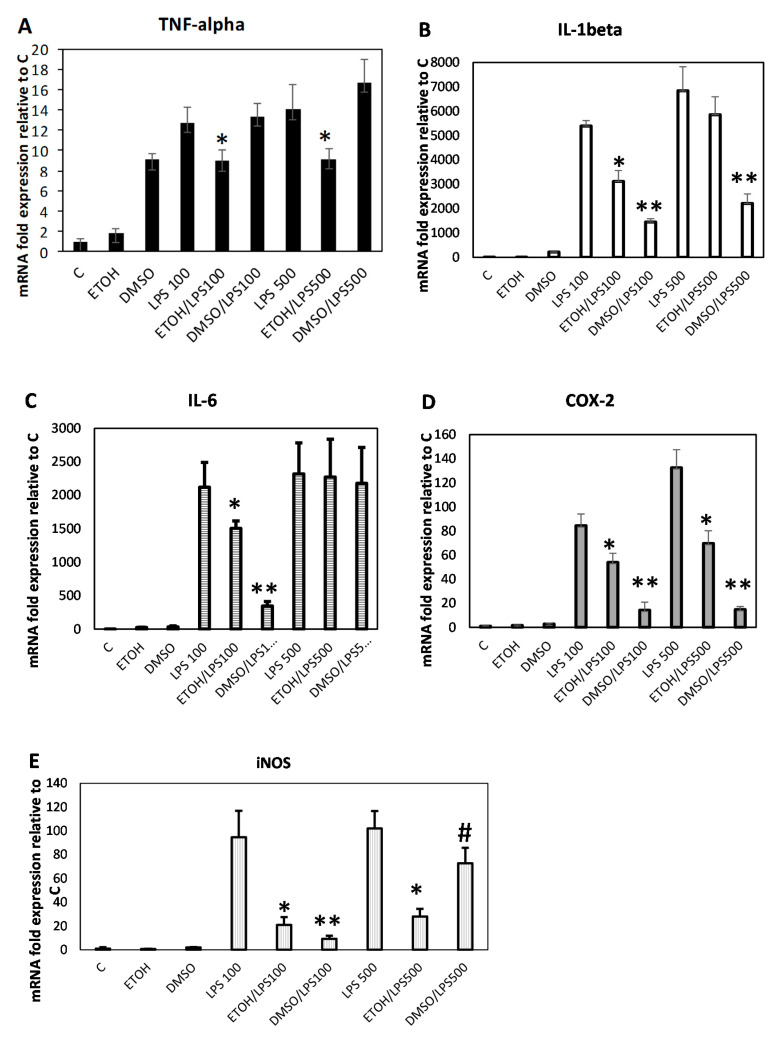
*amentacea* extract inhibition of gene expression in LPS-stimulated RAW 264.7 macrophages. Gene expression measured by qPCR analysis of TNF-alpha (**A**), IL-1β (**B**), IL-6 (**C**), COX-2 (**D**) and iNOS (**E**) after RAW 264. 7 cell incubation, for 8 h, with or without increasing concentrations of LPS (100-500 ng/mL) and in presence of 100 μg/mL 50%-ethanol extract or of 50 μg/mL of DMSO extract. Data are normalized on the GAPDH housekeeping gene and expressed as mRNA fold increase compared to control, untreated cells (in the presence of vehicle). Results are the mean ± SD of three experiments performed in triplicate. Asterisks indicate significance in Tukey test (TNF-alpha ANOVA *p* < 0.00001, Tukey vs. the respective LPS concentration, * *p* < 0.005; IL-1beta ANOVA *p* < 0.00001, Tukey vs. the respective LPS concentration, * *p* < 0.001; ** *p* < 0.0005, respectively; IL-6 ANOVA *p* < 0.0001, Tukey vs. the respective LPS concentration, * *p* < 0.05; ** *p* < 0.005, respectively; COX-2 ANOVA *p* < 0.00001, Tukey vs. the respective LPS concentration, * *p* < 0.01; ** *p* < 0.0001, respectively; iNOS ANOVA *p* < 0.00001, Tukey vs. the respective LPS concentration, # *p* < 0.05; * *p* < 0.005; ** *p* < 0.001, respectively).

RAW 264.7 macrophages were stimulated with increasing concentrations of the highly pro-inflammatory bacterial endotoxin (“LPS” bars, from 100 to 1000 ng/mL) and the NO production was evaluated after incubation in the presence or absence of the two *C. amentacea* extracts (Figure 6C). At the same time, the overexpression of inflammatory cytokines, Tumour Necrosis Factor-α (TNF-α), Cyclooxygenase-2 (COX-2), inducible NO synthase (iNOS), Interluekin-1β (IL-1β) and interleukin-6 (IL-6), was evaluated by quantitative PCR (qPCR, Figure 7) to finally assess the real anti-inflammatory potential of the two concoctions. After 24 h, LPS stimulation lead to a significant NO overproduction in RAW 264.7 macrophages at all concentrations of endotoxin used (Figure 6C, 79.3, 39.1 and 23.1 nmol/mL/mg protein for stimulation with LPS 1000, 500 and 100 ng/mL, respectively) compared to control cells (*p* < 0.0001 for the three LPS concentrations) in which the production was almost undetectable (Figure 6C, “C” bar). This NO production was completely inhibited by both ethanolic and DMSO extracts when LPS was used at the lowest concentration (“ETOH/LPS100” and “DMSO/LPS100” bars versus “LPS100” bar, respectively, *p* < 0.0001 for both). Furthermore, the NO overproduction was strongly inhibited by the two extracts in presence of the intermediate LPS concentration (500 ng/mL, “LPS500” bar vs. “ETOH/LPS500” bar, 81.7% inhibition, and versus “DMSO/LPS500” bar, 77.8% inhibition, respectively, *p* < 0.0001 for both). Finally, at the highest LPS concentration (1000 ng/mL) only the DMSO extract retained the ability to drastically inhibit the NO production in RAW 264.7 macrophages (“LPS1000” bar vs. “DMSO/LPS1000” bar, 78% inhibition, *p* < 0.0001), while the ethanolic extract only exhibited a slight, but still significant NO reduction (6.3% inhibition, “ETOH/LPS1000” bar vs. “LPS1000”, *p* < 0.0001). To our knowledge, this is the first demonstration that the extracts from an alga of the *Cystoseira* genus are capable of scavenging the natural, and potentially dangerous, direct production of NO by activated macrophages, again indicating a major anti-inflammatory effect in a more physiological setting like a cell culture, with respect to a cell-free spectrophotometric assay, such as the nitroprusside one, usually used in other studies [42].

Finally, we investigated the inhibition of gene expression upregulation of well-known inflammatory markers, after LPS stimulation of RAW 264.7 macrophages, in the presence of the two extracts, and also in this case, we could observe a dramatic anti-inflammatory effect of the *C. amentacea* derived mixture products towards IL1-β, IL-6, iNOS and COX-2 upregulation, as well as a partial inhibitory effect on TNF-α overexpression (Figure 7). In particular, the TNF-α mRNA was overexpressed by 12.7-fold and 14-fold after 8 h stimulation with LPS 100 and 500 ng/mL compared to control cells, respectively (Figure 7A, “LPS100” and “LPS500” bars vs. “C”, *p* < 0.005 for both). This overexpression was partially inhibited only by the ethanolic extract by 29.8 and 34.7% at the two LPS concentrations, respectively (“ETOH/LPS100” and “ETOH/LPS500” bars, *p* < 0.005 for both, compared to the respective LPS). Conversely, for the DMSO extract, it was not possible to observe any TNF-α overexpression inhibition at both LPS concentrations used. That was probably a consequence of the property of the DMSO extract that per se increases significantly the TNF-*α* expression by 9.1 folds compared to control cells (“DMSO” bar versus “C”). Concerning IL-1β upregulation, in the presence of LPS, a strong induction of gene expression was observed in RAW 264.7 macrophages, at both LPS concentrations used (Figure 7B, 5386-fold increase for “LPS100” and 6833-folds for “LPS500”, respectively, *p* < 0.0001 for both, compared to the control). This increase was significantly inhibited by both the ethanolic and DMSO extracts at the lowest LPS concentration, by 42%, and 73%, respectively (“ETOH/LPS100” and “DMSO/LPS100” bars vs. “LPS100”, *p* < 0.001 and *p* < 0.0005, respectively), while at the highest LPS concentration, only the DMSO extract retained the ability to inhibit the expression of this cytokine by 67.7% (“DMSO/LPS500” vs. “LPS500”, *p* < 0.0005). IL-6 strong upregulation, in the presence of LPS, was observed in RAW 264.7 macrophages, at both LPS concentrations used (Figure 7C, 2120-fold increase for “LPS100” and 2317-folds for “LPS500” *p* < 0.005 and *p* < 0.0001, compared to C, respectively). This increase was significantly inhibited by both the ethanolic and DMSO extracts at the lowest LPS concentration, by 29% and 84% respectively (“ETOH/LPS100” and “DMSO/LPS100” bars vs. “LPS100”, *p* < 0.05 and *p* < 0.005, respectively), while at the highest LPS concentration, none of the extracts were able to affect IL-6 overexpression. Since the three abovementioned cytokines, TNF-α, IL-1β and IL-6 are important inflammatory mediators, propagating the signals and recruiting more inflammatory cells, their significant inhibition at the mRNA level in our in vitro inflammatory simulator test by use of the *C. amentacea* extracts, suggests promising effects also in in vivo settings where the goal is always to abate these signals in order to resolve the inflammatory state [4,5,6]. Another important mediator of inflammation is PGE_2_, produced by the inducible enzyme cyclooxygenase-2 (COX-2). This molecule is responsible of many outcomes of acute inflammation like vasodilatation, oedema, influx of neutrophils and macrophages at the site of inflammation, increase of pain sensory response, and pyrogenic effect [53]. Thus, inhibition of PGE_2_ production is the main target of numerous anti-inflammatory drugs named NSAIDS (nonsteroidal anti-inflammatory drugs). These products in the years have shown many undesired side effects, thus there is a need in the pharmaceutical market of new, safer products, with selective action and lower toxicity which can be obtained from plants and isolated phytoconstituents [54]. The investigation of COX-2 mRNA expression in our in vitro inflammatory simulator test showed that both LPS concentrations were able to strongly upregulate this important inflammatory mediator, by 84.3 folds and 132.5 folds compared to control cells (Figure 7D, “LPS100” and “LPS500” bars vs. “C”, respectively, *p* < 0.0001 for both). At both LPS concentrations, both *C. amentacea* extracts were able to inhibit COX-2 mRNA synthesis. In particular, at 100 ng/mL LPS stimulation there was a significant inhibition of COX-2 upregulation by 36% in presence of the ethanolic extract and by 83% in presence of the DMSO extract (“ETOH/LPS100” and “DMSO/LPS100” bars vs. “LPS100”, *p* < 0.01 and *p* < 0.0001, respectively). At 500 ng/mL LPS stimulation, the two extracts inhibition was of 47.3% for the ethanolic and of 88.8% for the DMSO extract, respectively (“ETOH/LPS500” and “DMSO/LPS500” bars vs. “LPS500”, *p* < 0.01, and *p* < 0.0001, respectively). This significant effect for COX-2 expression again indicates the possibility to use these extracts as efficient anti-inflammatory drugs. Furthermore, since the effect is at the level of the mRNA synthesis, many of the side effects of traditional NSAIDS could be probably avoided by use of *C. amentacea* extracts. In fact, NSAIDS usually act as cyclooxygenase enzyme inhibitors acting both on COX-1 and COX-2 isoforms, and negative effects are usually due to inhibition of COX-1 isoform physiological role [54], which in the case of our extracts, would not be affected. Finally, also iNOS overexpression, responsible for the high levels of the cytotoxic NO radical in macrophages, was investigated. In RAW 264.7 cells both LPS concentrations were able to strongly upregulate this important enzyme, by 94.6 folds and 102.1 folds compared to control cells (Figure 7E, “LPS100” and “LPS500” bars vs. “C”, *p* < 0.001 and *p* < 0.0001, respectively). Even in this case, both extracts were able to inhibit overstimulated iNOS mRNA synthesis. In fact, at 100 ng/mL LPS stimulation there was a significant inhibition of iNOS by 78% with the ethanolic extract and by 90% with the DMSO extract (“ETOH/LPS100” and “DMSO/LPS100” bars vs. “LPS100”, *p* < 0.005 and *p* < 0.001, respectively). Conversely, at 500 ng/mL LPS stimulation, the ethanolic extract was able to inhibit iNOS overexpression by 73% and the DMSO extract by 29% (“ETOH/LPS500” and “DMSO/LPS500” bars vs. “LPS500”, *p* < 0.005 and *p* < 0.05, respectively). These data on iNOS inhibition are particularly interesting since they demonstrate for the first time that, other than a direct antioxidant effect exerted by scavenging the excess of NO produced by this enzyme, the *C. amentacea* extracted metabolites can also inhibit the preceding iNOS mRNA synthesis and protein production, de facto annihilating NO damage potential.

The fact that *C. amentacea* extracts exerts these significant inhibitory effects on so numerous essential inflammatory mediators, such as TNF, cytokines, prostaglandin synthase and nitric oxide synthase enzymes, suggests that the action of the extracts may be upstream of the main signal transduction pathways, leading to macrophage activation and a consequent change of gene expression profiles. One of the main, and well demonstrated, actions of the extracts is a potent ROS scavenging activity in cell-free as well as in cellular models (see Figure 1, Figure 2 and Figure 4). Since various ROS such as hydroxyl radical, superoxide anion and hydrogen peroxide, produced immediately after inflammatory stimuli by NADPH oxidase activation, are considered upstream signals able to activate both NF-κB as well as the MAPK signaling [55,56] responsible for the majority of downstream cell inflammatory response, the scavenging of ROS production by *C. amentacea* extracts in the cells at the very beginning of the inflammatory response is likely the reason for the strong inhibitory effects on macrophage activation in our in vitro inflammatory simulator test. 

The only data reported in the literature, showing an anti-inflammatory effect of extracts of the *Cystoseira* genus on a physiological model of inflammation, are those from Mhadhebi et al. [34] where *C. compressa*, *C. crinita* and *C. sedoides* aqueous extracts showed a beneficial effect, similar to dexamethasone treatment, on the rat paw oedema test, confirming the potential of extracts of the same genus also in an in vivo setting, although in that case the cellular and molecular mechanism of action of the extracts was not investigated.

## 3. Conclusions

The data obtained in this study demonstrate the strong anti-inflammatory effect of two *C. amentacea* extracts (DMSO and ethanolic) from the Ligurian Sea (North-western Mediterranean), by molecular and cellular analyses. The extracts showed a plethora of antioxidant and anti-inflammatory effects that were, for the first time, thoroughly investigated in this study by cell-free spectrophotometric tests, but most importantly, by the use of cellular models of toxicity and inflammation. The pleiotropic effects of the extracts in the well-known inflammatory model of LPS-stimulated RAW 264.7 macrophages point out the capacity of the metabolites, contained in the extracts, to act at different levels of the inflammatory process, both by abating the respiratory burst leading to the excess ROS production typical of the initial phase of the inflammatory response by immune cells, and also in the following phase, by blocking the production and release of important mediators propagating and exacerbating the process. Furthermore, the low cellular toxicity demonstrated by the two extracts opens the way to the use, in the near future, of its bioactive principles also for the formulation of antioxidant nutraceutical concoctions for anti-aging purposes as well as of cosmetic lotions for lenitive and restorative treatments to cure skin inflammatory states.

## 4. Materials and Methods

### 4.1. Chemicals

Here is the list of all reagents used: Dimethyl sulfoxide (DMSO), Ethanol, Folin-Ciocalteu reagent, Sodium Carbonate (Na_2_CO_3_), Gallic Acid, Aluminium Chloride (AlCl_3_), Sodium Nitrite (NaNO_2_), Sodium Hydroxide (NaOH), Quercetin, Formic Acid (FOA), Methanol, 2,2-diphenyl-1-picrylhydrazyl (DPPH), Phosphate buffer, Potassium Ferricyanide (K_3_[Fe(CN)_6_]), Trichloroacetic Acid (TCA), Ferric Chloride (FeCl_3_), Ascorbic Acid, Ethylenediaminetetraacetic acid (EDTA), Ferrous Ammonium Sulphate ((NH_4_)_2_Fe(SO_4_)_2_), Ammonium Acetate, Acetic Acid, Acetylacetone, Sodium Nitroprusside, Sulphanilamide, Naphthyl-ethylene-diamine, Orto-phosphoric Acid (H_3_PO_4_), Dulbecco’s modified Eagle’s medium (D-MEM), Glutamine, Fetal Bovine Serum (FBS), Penicillin, Streptomycin, 3-(4,5-dimethylthiazol-2-yl)-2,5-diphenyltetrazolium bromide (MTT), Hydrogen Peroxide (H_2_O_2_), Bacterial Lipopolysaccharide (LPS), 1,4-Dithiothreitol (DTT), Sodium Dodecyl Sulphate (SDS), Glycerol, Tris(hydroxymethyl)aminomethane hydrochloride (TRIS-HCl), Hank’s Balanced Salt Solution (HBSS), 2′,7′-dichloro-dihydro-fluorescein diacetate (DCF).

All reagents were acquired from SIGMA-ALDRICH (Milan, Italy), unless otherwise stated.

### 4.2. Algae Collection

In the Ligurian Sea (Northwestern Mediterranean), fronds of *Cystoseira amentacea* were collected in the midlittoral zone, on exposed rocky shores, at Bogliasco, Genoa (NW Italy, 44 2°2′40.37″ N—9°4′35.14″ E). The collection was performed in summer 2017, when the sea temperature values oscillated between 25 and 26°C. After collection, fronds were stored in plastic bags kept in cold conditions and immediately transported to the laboratory at the University of Genova.

### 4.3. Production of Extracts from Cystoseira amentacea

*C. amentacea* fronds were washed with deionized water, air-dried and cut to tiny pieces, and then dried in a lyophilizer. The extracts were obtained by incubation in mild conditions for 48 h in the dark in a rotary disk shaker at 30 °C of 2 g of lyophilized fronds in 20 mL of two different solvents:

- Dimethyl sulfoxide (DMSO)
- 50% ETHANOL

At the end of the extraction the suspensions were filtered with a strainer, an aliquot was lyophilized and weighted to determine the quantity of molecules extracted with the two solvents and finally they were diluted to a starting concentration of 5 mg/mL. Storage was done at −20 °C.

### 4.4. Total Phenolic Content

The total phenolic content (TPC) was determined according to the Folin-Ciocalteu assay reported by Biju et al. [57]. Briefly, the reaction mixture contained 100 μL of *C. amentacea* extracts (5 mg/mL starting concentration), 800 μL of deionized water and 100 μL of Folin-Ciocalteu phenol reagent. After 5 min of incubation at R.T., 1 mL of 10% Na_2_CO_3_ solution was added to the mixture. After incubation for 60 min in the dark at R.T, absorbance at 550 nm was read with a Beckman spectrophotometer (DU 640). Phenolic concentration was obtained by comparison with a calibration curve based on different concentrations of gallic acid (0.05 to 20 μg/mL), and the total phenolic content was expressed as mg Gallic acid Equivalents (GAE).

### 4.5. Total Flavonoid Content

Total flavonoid content (TFC) was measured by the AlCl_3_ colorimetric assay reported by Biju et al. [57]. The test solution was prepared with 100 μL of *C. amentacea* extracts (5 mg/mL starting concentration), 400 μL of distilled water and 30 μL of 5% NaNO_2_. After 5 min incubation at R.T., 30 μL of 10% AlCl_3_ were added, and after 5 more minutes, 200 μL of 1 M NaOH was added. Finally, the volume was made up to 1 mL with deionized water and absorbance was measured at 510 nm using a Beckman spectrophotometer (DU 640). Flavonoid concentration was obtained by comparison with a calibration curve based on different concentrations of quercetin (15 to 300 μg/mL), and the total flavonoid content was expressed as mg Quercetin Equivalents (QE).

### 4.6. HPLC-MS Analysis

The chromatographic separation of the two extracts was carried out by means of the Agilent 1100 µHPLC equipped with an automatic micro-sampler and a Symmetry C18 column (300 Å pore size, 5 µm particle size, 1 mm id × 150 mm) maintained at 30 °C. The injection volume was 8 µL. The chromatographic method consisted of the following gradient of 45 min: 0–5 min 8% B, 5–40 min 100% B, 40–45 min 100% B, at a flow rate of 30 µL/min, where A is H_2_O containing 1% FOA and B is methanol. The detector was set at 220/280 nm. Finally, the HPLC was coupled with the mass spectrometer (HPLC-ESI-MS) to qualitatively evaluate the compounds in the extract.

The instrument used is a mass spectrometer with an electrospray ion source (ESI) and a high capacity ion trap (Agilent 1100 MSD XCT ion trap). All parameters have been established to obtain the best ionization of the components. The analysis was performed in an ion charge mode control with target selected at 100,000 and accumulation time of 300 ms. The operating parameters were, capillary voltage: 3.6 V; nebulizer pressure: 20 psi; drying gas: 10 L/min; dry temperature: 350 °C; moving averages: 3, fragmentation width 1 V.

All mass spectra were acquired in full-scan and MS-MS mode, acquiring the most abundant species under each peak. Acquisition was performed on negative and positive ions in the 100–1000 mass range and analyzed using the integrated Agilent Data Analysis software (LC/MSD Trap Software).

### 4.7. DPPH Radical Scavenging Activity

The radical scavenging activity was evaluated by the DPPH method [58].

The DPPH test solution, 1 mL/sample, was prepared as follows: *C. amentacea* extract dilutions (1.25, 0.5 and 0.25 mg/mL) in 250 μL of water, 500 μL of methanol, 250 μL of 0.2 mM DPPH (2,2-diphenyl-1-picrylhydrazyl, Calbiochem^®^, Millipore SpA, Milan, Italy) dissolved in methanol.

In the blank sample, the DPPH solution was substituted with methanol, and a negative (only DPPH solution) and a positive control (ascorbic acid 0.5 mg/mL, final concentration) were made as well. All samples were incubated for 30 min at RT in the dark. Samples were read at 517 nm using a Beckman spectrophotometer (DU 640).

Due to the natural color of the extracts, a solution containing only the extracts in water:methanol 1:3 without DPPH was made as well, in order to subtract the natural absorbance of the extracts at 517 nm and correct the antioxidant scavenging values obtained. The procedure was carried out three times in duplicate.

### 4.8. Reducing Fe (III) Power Assay

The ability of *C. amentacea* extracts to reduce iron (III) was determined using the method reported by Sampath Kumar et al. [59]. *C. amentacea* dilutions (1.25, 0.5 and 0.25 mg/mL) of both types of extracts were mixed to 250 μL of 200 mM phosphate buffer (pH 6.6) and 250 μL of 1% potassium ferricyanide. The mixture was incubated at 50 °C for 30 min, and then 250 μL of 10% (*w*/*v*) TCA was added. The mixture was then centrifuged at 10,000 rpm for 10 min. 500 μL of the supernatant were mixed with 500 μL of distilled water and 100 μL of 0.1% (*w*/*v*) FeCl_3_. After 10 min the absorbance of the resulting solution was measured at 700 nm using a Beckman spectrophotometer (DU 640). The procedure was carried out in duplicate.

The negative control was produced by replacing the extracts with water, while in the positive control consisted of 20 μg/mL ascorbic acid. The reducing power was calculated as a percentage of the maximum activity of the positive control (20 μg/mL ascorbic acid).

### 4.9. OH Scavenging Activity

Hydroxyl radical scavenging activity of the extracts was determined using the Mohr’s salt method reported by Pavithra et al. [60]. The reaction mixture contained an aliquot of 100 μL of extract (starting concentration 5 mg/mL), 100 μL of iron-EDTA solution (0.13% ferrous ammonium sulphate 0.26% EDTA), 50 μL of 0.018% EDTA solution, 100 μL of DMSO solution (0.85% in 0.1 M phosphate buffer, pH 7.4) and 50 μL of 0.22% ascorbic acid. After an incubation at 80–90 °C for 15 min, the reaction was stopped by adding 100 μL of ice-cold TCA (17.5%). Finally, 300 μL of Nash reagent (75.0 g of ammonium acetate, 3.0 mL of glacial acetic acid and 2.0 mL of acetyl acetone in 1 L of distilled water) were added and read at 412 nm in a Beckman spectrophotometer (DU640) after 15 min. The intensity of the yellow color was measured at 412 nm against a blank reagent made with 300 μL of Nash reagent, 100 μL of ice-cold TCA (17.5%) and 600 μL of deionized water. The negative control was done by replacing the extracts with water, while in the positive control sample quercetin was used.

### 4.10. NO Scavenging Activity

NO-scavenging activity was evaluated using the assay reported by Xu et al. [61]. Various dilution of *C. amentacea* extracts (starting concentration 5 mg/mL) in 250 μL phosphate buffer (0.1 M, pH 7.4) were added to 500 μL of 5 mM sodium nitroprusside and 250 μL of phosphate buffer (pH 7.4). Samples were incubated under the light of a lamp at R.T. for 30 min, to produce NO. Afterwards, an equal volume of Griess reagent (1% sulphanilamide, 0.1% naphthyl-ethylene-diamine in 5% H_3_PO_4_) was added to the mixture. After incubation at R.T. for 10 min in the dark, the absorbance was measured at 546 nm trough a Beckman spectrophotometer. For the calibration curve, NaNO_2_ scalar dilution were used (1-5-10-50 μM). 

### 4.11. Cell Cultures

The mouse macrophage cell line RAW 264.7 and the mouse fibroblast L929 cell line were obtained from the American Type Culture Collection (LGC Standards srl, Milan, Italy). Cells were cultured at 37 °C in a humidified, 5% CO_2_ atmosphere in high glucose D-MEM with glutamine (Microtech srl, Naples, Italy), supplemented with 10% FBS (Microtech) with penicillin/streptomycin as antibiotics.

### 4.12. Cytotoxicity Assessment

The cytotoxicity of the extracts was evaluated on RAW 264.7 murine macrophages and L929 murine fibroblasts. RAW 264.7 macrophages were seeded at 25,000 cells/well, while the L929 fibroblasts were seeded at 10,000/well in 96-well plates. After one day, the extracts were added to the wells and the plates were incubated for 24 h at 37 °C. Experiments were performed in quadruplicate, *C. amentacea* extracts were added at 5, 10, 50 and 100 μg/mL final concentrations. Solvents alone were tested too (1% final dilutions). At the end of the incubation time, cell viability was assayed by the MTT test performed as already reported [62]. 

### 4.13. Rescue from H_2_O_2_ Cytotoxicity

Rescue by *C. amentacea* extracts from H_2_O_2_ cytotoxicity was evaluated in RAW 264.7 macrophages and L929 fibroblasts. Experiments were performed in quadruplicate on 96-well plates, RAW 264.7 macrophages were seeded at 25,000 cells/well, while the L929 fibroblasts were seeded at 10,000/well. After one day, cells were challenged with H_2_O_2_ 500 μM in the presence or absence of various concentrations of extracts (from 1 to 100 μg/mL), and all the plates were incubated for 24 h at 37°C. At the end of the incubation time, cell viability was assayed by MTT test.

### 4.14. Scavenging of NO Production in LPS-Treated RAW 264.7 Macrophages

RAW 264.7 macrophages were seeded at 1 × 10^6^ cells/well in 6-well plates, in duplicate. The day after cells were challenged with bacterial lipopolysaccharide (from 100 ng/mL to 1 μg/mL) in the presence or absence of *C. amentacea* extracts (50 and 100 μg/mL). After 24 h incubation at 37 °C the nitrite content of the cell media was quantified by the Griess assay (see above “NO scavenging activity” paragraph), while cells were lysed in 400 μL lysis buffer (100 mM DTT, 2% SDS, 10% glycerol and 50 mM TRIS-HCl, adjusted to pH 6.8). The lysates were heated at 100 °C for 10 min and the protein concentration was determined by the Bradford assay [63]. Nitrite production in each sample was then calculated through a standard NaNO_2_ curve and normalised on the protein content of the wells. The experiments were repeated three times.

### 4.15. ROS Intracellular Detection

Experiments were performed as already described [64]. Briefly, cells were seeded in quadruplicate on 96-well plates. RAW 264.7 macrophages were plated at a density of 25,000 cells/well while L929 cells at a density of 10,000 cells/well and allowed to adhere overnight. Cells were then washed once with HBSS and incubated for 30 min at 37 °C with 10 μM DCF dye (Life Technologies). After incubation with the dye, cells were washed with HBSS, incubated at 37 °C for 15 min and then challenged with 200 μM H_2_O_2_ for 2 h. The plates were finally read on a plate reader, Fluostar Optima BMG, using 485/520 excitation/emission wavelengths. Data are means ± S.D. of three independent experiments performed in quadruplicate.

### 4.16. Gene Expression Analysis in LPS-Activated RAW 264.7 Macrophages

RAW 264.7 macrophages were seeded at 1 × 10^6^ cells/well in 6-well plates, in duplicate. The day after cells were challenged with bacterial lipopolysaccharide (from 100 ng/mL to 500 ng/mL) in the presence or absence of *C. amentacea* extracts (50 and 100 μg/mL) for 8 h. Gene expression of the inflammatory mediators, tumor necrosis factor-alpha (TNF-α a.n. NM_001278601.1), interleukin-1beta (IL-1β a.n. NM_008361.4), interleukin-6 (IL-6, NM_031168.2), inducible NO synthase (iNOS, NM_010927.4) and cyclooxygenase-2 (COX-2, a.n. NM_011198.4) normalised on GAPDH housekeeping gene (a.n. NM_001289726.1) was evaluated by qPCR. Cell RNA was extracted using the RNeasyMini Kit (Qiagen, Milan, Italy), according to the manufacturer’s instructions. Quality and quantity of RNA was analysed using a NanoDrop spectrophotometer (Nanodrop Technologies, Wilmington, DE, USA). The cDNA was synthesized from 1 μg RNA by using iScript cDNA Synthesis Kit (Bio-Rad Laboratories, Milan, Italy). Each PCR reaction was performed in 10 μL containing: 1 × master mix iQ SYBR Green (Bio-Rad), 0.2 μM of each primers and 5 ng of synthesized cDNA. All samples were analysed in triplicate. The following thermal conditions were used: Initial denaturation at 95 °C for 3 min, followed by 45 cycles with denaturation at 95 °C for 15 s, annealing and elongation at 60 °C for 60 s. The fluorescence was measured at the end of each elongation step. The values were normalized to GAPDH (reference gene) mRNA expression. All primers (Table 2) were designed using the Beacon Designer 7.0 software (Premier Biosoft International, Palo Alto CA, USA) and obtained from TibMolBiol (Genova, Italy). Data analyses were obtained using the DNA Engine Opticon 3 Real-Time Detection System Software program (3.03 version) and, in order to calculate the relative gene expression compared to an untreated (control) calibrator sample, the comparative threshold Ct method was used [65] within the Gene Expression Analysis for iCycler iQ Real Time Detection System software (Bio-Rad, Milan, Italy).

### 4.17. Statistical Analyses

Statistical analyses were performed through one-way ANOVA and Tukey’s post-test per each extract, in order to assess possible differences among the various concentrations used in each assay (GraphPad Software, Inc., San Diego, CA, USA). *p* < 0.05 were considered significant.

## Figures and Tables

**Table 1:** TPC and TFC of *C. amentacea* extracts.

Type of Extract	TPC (μg/mg Crude Extract)	TFC (μg/mg Crude Extract)
50%-ethanol	20.3 ± 0.74	3.1 ± 0.48
DMSO	65.9 ± 1.74	15.8 ± 0.51

Quantification of the total phenolic content (TPC) and of the total flavonoid content (TFC) by the Folin-Ciocalteu and by the AlCl_3_ colorimetric assays, respectively in the two extracts obtained by *C. amentacea*. Values are expressed as mean ± S.D.

**Table 2:** List of primers used in qPCR experiments.

GENE	GenBank (a.n.)	Forward	Reverse	Size (bp)
COX-2	NM_011198.4	CCAgCAAAgCCTAgAgCAAC	AgCACAAAACCAggATCAgg	126
IL-1β	NM_008361.4	gCAgCACATCAACAAgAg	CAgCAggTTATCATCATCATC	184
TNF-α	NM_001278601.1	CCACCATCAAggACTCAA	ATCTTATCCAgCCTCATTCT	120
IL-6	NM_031168.2	ACCTgTCTATACCACTTC	gCATCATCgTTgTTCATA	117
iNOS	NM_010927.4	CCgCCgCTCTAATACTTA	TTCATCAAggAATTATACAggAA	121
GAPDH	NM_001289726.1	TCTCCCTCACAATTTCCATCCCAg	gggTgCAGCgAACTT TATTgATgg	99

Primer pairs used in gene expression quantification by qPCR analysis in RAW 264.7 murine macrophages.
